# Flares in patients with systemic lupus erythematosus

**DOI:** 10.1093/rheumatology/keaa777

**Published:** 2020-12-16

**Authors:** Kathleen McElhone, Janice Abbott, Margaret Hurley, Jane Burnell, Peter Lanyon, Anisur Rahman, Chee-Seng Yee, Mohammed Akil, Ian N Bruce, Yasmeen Ahmad, Caroline Gordon, Lee-Suan Teh

**Affiliations:** 1 Department of Rheumatology, Royal Blackburn Hospital, Haslingden Road, Blackburn; 2 School of Psychology; 3 Faculty of Health and Wellbeing, University of Central Lancashire, Preston; 4 Rheumatology, Nottingham University Hospitals NHS Trust; 5 Epidemiology and Public Health, University of Nottingham, Nottingham; 6 NIHR Nottingham Biomedical Research Centre, Nottingham; 7 Centre for Rheumatology Research, University College London, The Rayne Building, 4th Floor, 5 University Street, London; 8 Department of Rheumatology, Doncaster Royal Infirmary, Armthorpe Road, Doncaster; 9 Department of Rheumatology, Royal Hallamshire Hospital, Glossop Road, Sheffield; 10 Arthritis Research UK Centre for Epidemiology, Faculty of Biology, Medicine and Health, The University of Manchester and NIHR Manchester Biomedical Research Centre, Manchester University Hospitals NHS Foundation Trust, Manchester Academic Health Science Centre, Manchester; 11 Peter Maddison Rheumatology Centre, Betsi Cadwaldr University Health Board, Llandudno Hospital, Llandudno, Conwy; 12 Rheumatology Research Group, Institute of Inflammation and Ageing, College of Medical and Dental Sciences, University of Birmingham; 13 Rheumatology Department, City Hospital, Sandwell and West Birmingham Hospitals NHS Trust; 14 NIHR/Wellcome Trust Birmingham Clinical Research Facility, University Hospitals Birmingham NHS Foundation Trust, Birmingham; 15 Faculty of Clinical and Biomedical Sciences, University of Central Lancashire, Preston PR1 2HE, UK

**Keywords:** flares, disease activity, BILAG-2004 index, SLE

## Abstract

**Objective:**

SLE is characterized by relapses and remissions. We aimed to describe the frequency, type and time to flare in a cohort of SLE patients.

**Methods:**

SLE patients with one or more ‘A’ or ‘B’ BILAG-2004 systems meeting flare criteria (‘new’ or ‘worse’ items) and requiring an increase in immunosuppression were recruited from nine UK centres and assessed at baseline and monthly for 9 months. Subsequent flares were defined as: severe (any ‘A’ irrespective of number of ‘B’ flares), moderate (two or more ‘B’ without any ‘A’ flares) and mild (one ‘B’).

**Results:**

Of the 100 patients, 94% were female, 61% White Caucasians, mean age (s.d.) was 40.7 years (12.7) and mean disease duration (s.d.) was 9.3 years (8.1). A total of 195 flares re-occurred in 76 patients over 781 monthly assessments (flare rate of 0.25/patient-month). There were 37 severe flares, 32 moderate flares and 126 mild flares. By 1 month, 22% had a mild/moderate/severe flare and 22% had a severe flare by 7 months. The median time to any ‘A’ or ‘B’ flare was 4 months. Severe/moderate flares tended to be in the system(s) affected at baseline, whereas mild flares could affect any system.

**Conclusion:**

. In a population with active SLE we observed an ongoing rate of flares from early in the follow-up period with moderate–severe flares being due to an inability to fully control the disease. This real-world population study demonstrates the limitations of current treatments and provides a useful reference population from which to inform future clinical trial design.

***Rheumatology* key messages**

- In this observational study, SLE patients flare early on in the follow-up period.
- The median time to any ‘A’ or ‘B’ flare (BILAG-2004 index) was 4 months.
- This real-world population study provides a useful reference which can inform future clinical trial design.

## Introduction

SLE is a major autoimmune multi-system rheumatic disease that is most common in women during the childbearing years [1]. It can affect any organ system and the disease varies in its clinical manifestations and severity between individuals. For most patients the disease is characterized by unpredictable relapses (flares) and remissions [2]. In recent years, mortality rates have improved [3] but there is still no cure, and flares of disease, infection and damage all continue to contribute to excess morbidity and mortality [4].

Flare is defined by International Consensus as *‘*a measurable increase in disease activity in one or more organ systems involving new or worse clinical signs and symptoms and/or laboratory measurements. It must be considered clinically significant by the assessor and usually there would be at least consideration of change or an increase in treatment’ [5]. Flares are assessed using various validated disease activity measures. However, there is no standardized definition of a measurable increase in disease activity. The three main disease activity indices currently used in clinical trials are the Safety of Estrogen in Lupus Erythematosus National Assessment Trial-SLEDAI (SELENA-SLEDAI) [6, 7], SLEDAI-2K [8] and the BILAG-2004 index [9]. For the SLEDAI and its derivatives, items are scored numerically and a numerical increase of at least three compared with the previous assessment constitutes a flare [5, 10, 11]. For the BILAG-2004 index, the presence of an item that is ‘worse’ or ‘new’ constitutes a flare [12–14].

Flares have been associated with more hospitalizations [15] and more organ and system damage, which in turn can lead to poorer prognosis and increased mortality [16–18]. In addition, the prolonged use of CS in the presence of persistence of disease or during flares can contribute to damage [19]. Flares, damage and prolonged use of CS can contribute to poor health-related quality of life [20–23]. Therefore, flare prevention is an important treatment goal in patient management.

Understanding the pattern of flares in SLE patients would be informative not only in the day to day management of these patients, but also in the interpretation of clinical trials of new medications where frequency and type of flares are included as outcome measures. This study describes the flare rates and types of flares in a prospective observational multicentre study of patients with active SLE after treatment of a flare.

## Methods

The study was granted Multicentre Research Ethics Committee (MREC 02/5/035) approval and participants from the collaborating centres gave written informed consent. The collaborating rheumatology units were UK centres with an interest in SLE as part of the BILAG: Bangor, Birmingham (two centres), Blackburn, University College London, Nottingham, Manchester, Doncaster and Sheffield.

The inclusion and exclusion criteria of the patients recruited, and the demographic and clinical data collected have been detailed previously in the longitudinal study to determine the sensitivity to change of the LupusQoL [24]. Patients were eligible to be included in the study if they had a flare of SLE requiring specific treatment. For this study, flare was defined as a significant increase in disease activity resulting in a BILAG-2004 index ‘A’ or ‘B’ score based on manifestation(s) that are ‘new’ or ‘worse’ [9, 13, 14, 25]. In addition, the flare definition for this study required patients to have an increase in therapy defined as one or more of the following: an increase of oral prednisolone to ≥20 mg/day, introduction of MTX, parenteral methylprednisolone, and/or other immunosuppressive therapy (e.g. CYC, rituximab). These patients were followed up monthly for 9 months and the BILAG-2004 disease activity index was assessed at each time point. For the purposes of this study, subsequent flares were defined as: severe (‘A’ flare/s irrespective of number of ‘B’ flares), moderate (two or more ‘B’ flares without any ‘A’ flares) and mild (one ‘B’ flare). We calculated the total numerical BILAG-2004 score at baseline where A = 12, B = 8, C = 1 for each system [25].

### Statistical methods

Patient data were summarized using the following descriptive statistics; means (s.d.), medians (interquartile ranges) and/or frequency counts. Flare rates were expressed as the number of flares per patient-month. Time to flare was also estimated using the Kaplan–Meier method.

## Results

### Patient characteristics

We recruited 100 patients with a mean (s.d.) age and disease duration of 40.7 (12.7) years and 9.3 (8.1) years, respectively. The study population consisted of 94% females, 62.6% White Caucasians, 15.2% south Asians, 8.1% Black Caribbean, 4% Black Africans, 5% mixed and 1% Chinese. At baseline (initial flare), the median (range) numerical BILAG-2004 score was 14 (10–21). The baseline characteristics are summarized in Table 1. Table 2 show the medications the patients were on at recruitment including for the treatment of the flare.

**Table 1:** Patient baseline demographic and clinical characteristics [*n* (%) unless stated]

Females	94 (94)
Mean (s.d.) age/disease duration (years)	40.7 (12.7)/9.3 (8.1)
Ethnic distribution (*n* = 99)	
White (British, Irish)	62 (63)
Black (Caribbean, African)	12 (12)
Asian (Indian, Pakistani, Bangladeshi)	14 (14)
Chinese	1 (1)
Other Asian	3 (3)
Mixed	7 (7)
Baseline clinical characteristics (ACR criteria)	
Malar rash	43 (43)
Photosensitivity rash	47 (47)
Discoid rash	12 (12)
Mouth ulcers	47 (47)
Arthritis	92 (91)
Serositis	45 (45)
Renal disease	21 (21)
CNS disease	8 (8)
Haematological disease	73 (72)
Positive ANA	96 (95)
Positive dsDNA, Sm or Antiphospholipid antibodies (APA) antibodies	80 (79)
BILAG-2004 index numerical score, median (range)	14 (10–21)

**Table 2:** Treatment at start of the study (for treatment of flares and background medications at recruitment)

Medications	*n* = 100^a^
AZA	17
CYC	6
HCQ	74
LEF	1
MTX	15
MMF	25
Rituximab	11
Steroids (oral or i.v. or i.m.)	87

a Most patients were on two medications and some were on three medications.

### Flare rates and types of flares

During follow-up, 195 flares occurred in 76 patients over 781 months of follow-up (0.25 flares per patient-month). Table 3 summarizes the flare severity category in these patients: there were 37 severe flares in 22 patients, 32 moderate flares in 19 patients and 126 mild flares in 67 patients. Twenty-nine patients had more than one type of flare in the 9 months: 12 had severe and mild flares, 14 had moderate and mild flares and three patients had all three types of flares (severe, moderate and mild). Twenty-four (24%) patients did not experience any ‘A’ or ‘B’ flares. The median time to any ‘A’ or ‘B’ flare was 4 months.

**Table 3:** Type and frequency of flares

Types of flares (using BILAG-2004 index) per patient (*n* = 100)	Number of patients (*n*)
Severe (any ‘A’ flares irrespective of ‘B’ flares)	
Any severe flare	22
Only one severe flare	15
Multiple severe flares	7
Severe flares only (without moderate/mild flares)	7
Severe and moderate flares only	0
Severe and moderate and mild flares	3
Severe and mild flares only	12
Moderate (two or more ‘B’ flares without any ‘A’ flares)	
Any moderate flare	19
Only one moderate flare	12
Multiple moderate flare	7
Moderate flares only (without severe/mild flares)	2
Moderate and severe flares only	0
Moderate and severe and mild flares	3
Moderate and mild flares only	14
Mild (one ‘B’ flare)	
Any mild flare	67
Only one mild flare	36
Multiple mild flares	31
Mild flares only (without severe/moderate flares)	38
Mild and severe flares only	12
Mild and severe and moderate	3
Mild and moderate flares only	14
No flares (no ‘A’ or ‘B’ scores due to items new or worse)	
Patients with no ‘A’ or ‘B’ scores	24

**Fig. 1:**
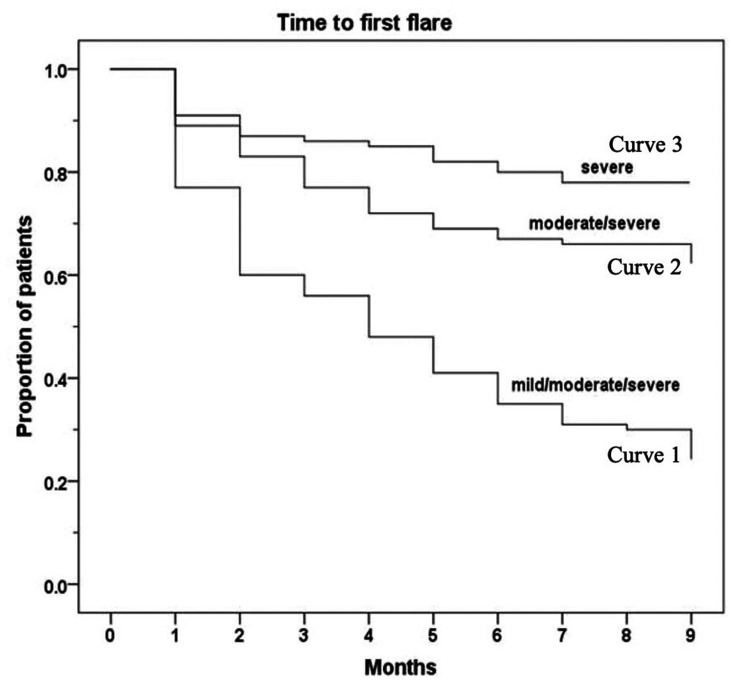
Time to first flare with regards to type of flares (mild, moderate or severe) Curve 1: time to first mild/moderate/severe flare. Curve 2: time to first moderate/severe flare. Curve 3: time to first severe flare.

By 1 month, 22% of all patients had a mild/moderate/severe flare; 22% of all patients had a moderate/severe flare by 3 months; and 22% of all patients had a severe flare by 7 months. Fig. 1 shows the time to the first mild/moderate/severe flare (Curve 1), moderate/severe flare (Curve 2) and severe flare (Curve 3).

The BILAG-2004 system(s) that had ‘A’ and/or ‘B’ flares at baseline were as follows: musculoskeletal (39.4%), mucocutaneous (21.8%), cardiorespiratory (13.5%), renal (11.8%), constitutional (7.7%), neuropsychiatric (2.4%), gastrointestinal (2.4%) and ophthalmic (1.2%). The systems affected at baseline were compared with those affected at the time of the first severe, moderate and mild flares. Severe (13.6% discordant) and moderate (5.3% discordant) flares tended to be in the same system(s) affected at baseline, whereas mild flares were more likely to affect any system (34.3% discordant) (Table 4).

**Table 4:** BILAG-2004 index system affected at baseline and time of flare and concordance between the two

BILAG-2004 index	At baseline (all patients) (%); *n*_pat_ = 100, *n*_flare_ = 100, *n*_system_ = 170	Severe flare (system with ‘A’ flare) (%); *n*_pat_ = 22, *n*_flare_ = 37, *n*_system_ = 38	Moderate flare (≥2 ‘B’ flares) (%); *n*_pat_ = 19, *n*_flare_ = 32, *n*_system_ = 75	Mild flare (1 ‘B’ flare) (%); *n*_pat_ = 67, *n*_flares_ = 126, *n*_system_ = 126
Systems affected				
Constitutional	13 (7.7)	2 (5.3)	2 (2.7)	2 (1.6)
Mucocutaneous	37 (21.8)	15 (39.5)	19 (25.3)	42 (33.3)
Neurological	4 (2.4)	2 (5.3)	7 (9.3)	8 (6.3)
Musculoskeletal	67 (39.4)	15 (39.5)	21 (28)	41 (32.5)
Cardiorespiratory	23 (13.5)	0	12 (16)	14 (11.1)
Gastrointestinal	4 (2.4)	0	5 (6.7)	1 (0.8)
Ophthalmic	2 (1.2)	2 (5.3)	0	0
Renal	20 (11.8)	2 (5.3)	9 (12)	18 (14.3)
Haematology	0	0	0	0
Concordance with baseline system/s affected at first flare per patient (% of patients)				
Complete concordance		19 (86.4)	9 (47.4)	44 (65.7)
Partial concordance		n/a	9 (47.4)	n/a
Complete discordance		3 (13.6)	1 (5.3)	23 (34.3)

*n*_pat_: number of patients; *n*_flare_: number of flares; *n*_system_: number of times system affected with flare; %: percentage of total number of systems affected with flare; n/a: not applicable.

## Discussion

This multicentre prospective observational study captured the frequency of subsequent flare in a population entering the study at the time of moderate or severe flare. Using the BILAG-2004 index, we have described the pattern of flares in patients with SLE who were treated for severe or moderate flares over a 9-month period. These patients were followed monthly and were treated with various medications. We found that 76% of patients had a subsequent flare and the flare rate was 0.25/patient-month, of which 19% were severe, 16% moderate and the rest were mild flares. The flares were predominantly in the original organ system, demonstrating the inadequacy of current treatment to control disease and to prevent further flares that increase the risk of future organ damage.

There are only a small number of studies in the literature exploring incidence of flare in SLE as an outcome. These studies are difficult to compare with our study or with each other for the following reasons; different study populations and various methodological differences including study design (observational/interventional), duration of study, outcome measures employed and flare definitions. The challenges of making cross study comparisons are illustrated in supplementary Table S1, available at *Rheumatology* online, in studies that have used the BILAG index as a disease activity measure and a similar definition of flare [26–31].

Our study presents real-world data on patterns of flares and flare frequency in SLE patients in the UK after treatment of a severe or moderate flare with conventional therapy [32]. Thus, the patients included in this study are typical of those that meet the eligibility criteria (inclusion criteria of moderate–severe flares) for an interventional study. The frequency (monthly) of patient review has generated a wealth of data not only on the natural history of subsequent flares after standard treatment (flare rates), but also on the types (severity and system involvement) of flares. Thus, the findings of our study may be of relevance to inform the design of interventional studies.

## Supplementary Material

keaa777_Supplementary_Data
