# Supplementary material for: Flares in patients with systemic lupus erythematosus
Source: Rheumatology (Oxford). 2020 Dec 16;60(7):3262–7. doi: 10.1093/rheumatology/keaa777 (PMC8517882; doi:10.1093/rheumatology/keaa777)
Supplement: keaa777_Supplementary_Data [file keaa777_Supplementary_Data.docx]

**Supplementary Table S1: Summary of findings regarding flares in SLE from studies utilising BILAG index (including current study)**

| **Author^Reference^** | **Measures used** | **Type of study/**  **Duration/No of centres** | **No of patients/**  **Intervention** | **Definition of Flares** | **Description of Flare results** |
| --- | --- | --- | --- | --- | --- |
| Current Study  McElhone *et al* | BILAG-2004 | Observational  Prospective  9 months  Multi-centre (n=9) | n=100  Various (not specified - study is observational) | BILAG scores based on new or worse items  Severe: ≥ 1 A  Moderate: ≥2 B  Mild: 1 B | 195 flares in 76 patients over 781 monthly. Flare rate of 0.25/month.  37 severe flares (22 patients), 32 moderate flares (19 patients) and 126 mild flares (67 patients).  Median time to any A or B flare was 4 months (95% CI 2.7 to 5.3).  Severe and moderate flares tend to be in the system/s affected at baseline whereas mild flares can affect any system. |
| Ehrenstein *et al^26^* | Classic BILAG | Observational  Prospective  Single centre  3 years | n=114  Various (not specified - study is observational) | Change in BILAG score of D or E to B or an increase to an A from any previous score | 458 Flares in 107 patients. Mean Incidence = 3 flares/patient/year  69% ≥ one flare  54% ≥ one system simultaneously, 24% flares one system, 6% no flares, 16% had flares in different systems at different times  70% only one system flare  “A” Flares most common in Musculoskeletal system  No mention of time to flare |
| Gordon *et al^12^* | Classic BILAG | Observational  2 centres  12 months | n=250  Various (not specified - study is observational) | New A or B score | 154 (61.6%) patients flared  A flare in 26 (10.4%), B flare from D/E in 65 (26%), B flare from C in 63 (25.2%)  No mention of time to flare |
| Merrill *et al^27^* | Classic BILAG | Phase IIb randomised  double-blind, placebo controlled trial  Multicentre  12 months | n=118 Abatacept  n=57 for placebo | New A or B score after steroid taper | 79.7% new A or B flare in abatacept group vs 82.5% in placebo group  40.7% A flares in abatacept group vs 54.4% A flares in placebo group  No mention of time to flare |
| Merrill *et al^28^* | Classic BILAG | Clinical trial  Multi-centre  52 week study | n=257 Intent to treat  Rituximab (n=169)  Placebo (n=88) | New or persistent A or B score and starting or increase steroid dose  Severe ≥ 1 A or ≥ 3 B  Moderate 2 B | Moderate or severe flares: 81/127 (64%) (RTX), 37/58 (64%) (Placebo)  Time to flare 3.7 months vs 4.1 months  Severe flares (≥ A or 3 Bs): (RTX vs placebo)  Time to flare 10.1 vs 7.3 months  When only ≥ A flares: 43/127 (34%) vs 27/58 (47%)  Mean annualised “A” flare rate: 0.86 vs 1.41 |
| Isenberg *et al^29^* | Classic BILAG | Double blind, placebo- controlled for Atacicept 150mg, 75mg and placebo 1:1:1 after control of A or B flare at 12/52 with steroids and  7.5 mg of Pred allowed at week 12/52  52 week study | n=461 randomised  n=455 received trial medication 75mg Atacicept  150mg atacicept discontinued due to 2 deaths | Flare ≥ one A or B due to items that were new or worse after significant improvement (not defined in paper but was 2 visits) | For the ITT group:  Flare rates: 58% for Atacicept 75mg, 54% placebo groups (37% atacicept 150mg)  Time to first flare no different between 75mg Atacicept and placebo but delayed in atacicept 150mg group  Between week 24 to 52, for those who completed the 24 weeks, 150mg Atacicept showed a reduction in flare rate of 16% compared to 36% in placebo group.  For the PC (potential completer) group:  Flare rates: 58% atacicept 75mg and 60% placebo (43% Atacicept 150mg)  Those on 150mg Atacicept had a 59% lower risk of having a new BILAG A and B flare compared to SOC and placebo group  Between week 24 to 52, for those who completed the 24 weeks, 150mg Atacicept showed a reduction in flare rate of 15% compared to 39% in placebo group.  There was also a reduction in the proportion of patients with a new flare in the eight systems in the 150mg Atacicept group compared to the other two groups. |
| Merrill *et al^30^* | *SRI-4 - SLE responder Index-4 - | phase IIb multicentre, randomized, double-blind, placebo-controlled  24-week | n=306  1:1:1  75mg, 150mg of Atacicept or placebo | Severe Flares defined by SELENA-SLEDAI flare index (SFI) or new BILAG A score | The incidence of A BILAG scores reduced with Atacicept 75mg  The atacicept 150mg group had fewer flares as defined by SFI  For the HAD (high disease activity) group at baseline, both doses of Atacicept led to reductions in severe flares by both definition |
| Furie *et al^31^* | *SRI | Continuation study of BLISS-76 clinical trial (Phase III randomised study  7 years | n=268 enrolled  n=140 completed  Belimumab 10mg/kg | SELENA-SLEDAI flare index and one BILAG new A or > 1 B score compared to baseline | At each yearly visit, ≥ 95% had no new BILAG A and no more than 1 new BILAG B.  At midpoint year one, 97.4% had no new BILAG A and no more than 1 new BILAG B.  At end of study year 7, 98.4% had no new BILAG A and no more than 1 new BILAG B  Of all the patients that flared, 12.4% and 31.5% had at least one BILAG flare by study year 1 and study year 7 midpoints respectively. |

*SRI = SLE responder index (Composite end point which includes SLEDAI score ≥ 4 point reduction, < 10% increase in PGA, no new BILAG 2004 A and no more than 1 new B)
